# Molecular sexing of degraded DNA from elephants and mammoths: a genotyping assay relevant both to conservation biology and to paleogenetics

**DOI:** 10.1038/s41598-021-86010-x

**Published:** 2021-03-31

**Authors:** Laetitia Aznar-Cormano, Julie Bonnald, Sabrina Krief, Nelson Guma, Régis Debruyne

**Affiliations:** 1 CNRS, Centre de Recherche en Paléontologie Paris (CR2P), Muséum national d’Histoire naturelle, Sorbonne Université, 57 rue Cuvier, CP 38, 75005 Paris, France; 2 CNRS, Eco-Anthropologie (EA), Muséum national d’Histoire naturelle, Université Paris Diderot, 17 place du Trocadéro, 75016 Paris, France; 3 Sebitoli Chimpanzee Project, Sebitoli Research Station, Kibale National Park, Fort Portal, Uganda; 4 Uganda Wildlife Authority, Kampala, Uganda; 5 Direction Générale Déléguée à la Recherche, à l’Expertise, la Valorisation et l’Enseignement (DGD-REVE), Muséum national d’Histoire naturelle, 57 rue Cuvier, CP 17, 75005 Paris, France

**Keywords:** Conservation biology, Evolutionary biology, Genetic variation, Genotype, Genetic markers

## Abstract

It is important to determine the sex of elephants from their samples—faeces from the field or seized ivory—for forensic reasons or to understand population demography and genetic structure. Molecular sexing methods developed in the last two decades have often shown limited efficiency, particularly in terms of sensitivity and specificity, due to the degradation of DNA in these samples. These limitations have also prevented their use with ancient DNA samples of elephants or mammoths. Here we propose a novel TaqMan-MGB qPCR assay to address these difficulties. We designed it specifically to allow the characterization of the genetic sex for highly degraded samples of all elephantine taxa (elephants and mammoths). In vitro experiments demonstrated a high level of sensitivity and low contamination risks. We applied this assay in two actual case studies where it consistently recovered the right genotype for specimens of known sex a priori. In the context of a modern conservation survey of African elephants, it allowed determining the sex for over 99% of fecal samples. In a paleogenetic analysis of woolly mammoths, it produced a robust hypothesis of the sex for over 65% of the specimens out of three PCR replicates. This simple, rapid, and cost-effective procedure makes it readily applicable to large sample sizes.

## Introduction

Molecular sexing has proven to be of use in a broad range of applications in biology, which encompass conservation biology, paleogenetics, and zooarcheology. For endangered species like modern elephants, the ability to monitor the sex distribution within and between populations is critical to ecological surveys and conservation programs^1^. For example, it has revealed an artificial bias in sex ratio in natural populations from different areas of the world, due to the selective poaching towards large tusk-bearing (i.e. male) elephants^2,3^. In areas where human-elephant conflicts occur, knowing the sex of the raiding individuals might help mitigating tensions, by integrating the known differences in the crop feeding behavior between males and females^4–6^.

In the course of the genetic surveys of wild populations of elephants, the most typical sampling material has long been non-invasive fecal samples^7–10^. The collection of such material generally happens without the actual sighting of the animal, so that its individual sex remains unknown. Since the size of the dung bolus is highly correlated to the actual size of the animal, it allows to recognize that the largest dung boli belong to male specimens, thanks to the important sexual dimorphism in elephants^11^. However, for the large majority of dung boli, the sex of the individual remains indiscernible. The other typical material used in elephants’ genetics is seized ivory^12,13^ for which the same rationale can be applied to determine the sex, hence with the same general limitations. Molecular methods have thus been sought to provide a quick and effective assessment of the sex of elephants. Yet, the nature of these samples entails another difficulty in their genetic analysis. Depending on the freshness of the dung collected, the elephant DNA content of a sample can vary extensively in amounts and degradation levels. After five days of deposition, the targeted PCR amplification of either mitochondrial or nuclear DNA has proven to be difficult to impossible^14^. Although the high mineral content of the ivory allows it to retain some DNA up to thousands of years in optimal conditions^15^, its low original content in living cells reduces the overall amount of DNA that can be recovered^16^. The DNA extracted from either ivory stocks or dung thus usually exhibits typical features of post-mortem degradation, in particular a high level of fragmentation. These features make the molecular determination of the sex from such samples non-trivial.

The range of scientists interested in the ability to determine the genetic sex of elephantine specimens extends beyond the scope of conservation biologists. The paleogeneticists who study the evolution of the extinct mammoths and elephant forms also have a keen interest in the sex of their specimens^17^. Discoveries of sub-complete specimens are exceptional, and although the size of individual bones might sometimes be sufficient to establish the sex of a specimen, most of the ancient elephantine remains cannot be accurately sexed through an anatomical analysis. Yet, this knowledge is key to directly address the social and ecological features of these extinct lineages, which are otherwise mostly inferred from actualistic comparisons where modern elephants’ biology serves as a template^18^. Because of the strong sexual bias that conditions the dispersion of elephantids—solitary male disperse whereas female-led herds exhibit a high level of philopatry^19^—knowing the sex of the specimens could also be useful to identify possible migrants. The extensive work published on mammoth paleogenomes in the last decade confirmed the advanced state of DNA degradation for these animals, even when preserved in the permafrost^20^, so that most of their endogenous DNA content is composed of fragments below 100 or even 50 nucleotides long. Although this preservation has allowed the sequencing of dozens of mammoth mitogenomes^21^, the direct amplification of mammoth nuclear DNA via conventional targeted PCR remains extremely difficult^22^. Until now, the only published method to establish the sex of an elephantine specimen for which ancient DNA is available has relied on a statistical analysis of the relative depth of coverage of the autosomes and the X chromosome to determine its valence^17,23^. This methodology is appealing in its own rights, as it reveals the sex as a by-product of the genome sequencing of these animals—even at moderate sequencing depth. Nevertheless, it is far too costly and cumbersome to be adopted as a standalone diagnostic test of the sex for elephantine specimens when large amounts of specimens are to be analyzed.

Until recently, the two main strategies developed for sexing mammals with PCR analysis have been applied to perform the molecular sexing of elephants. The first strategy relies on the targeted PCR amplification of a molecular marker present on both X and Y chromosomes and bearing sex-specific allelic features. It uses the conservation of the sequence at the regions flanking the polymorphic sites to design a single primer pair in order to co-amplify both alleles—and avoid potential biases due to uneven sensitivity. It then uses the inner-sequence variation to genotype the X and Y alleles separately. In their endeavour to generalize this approach among various mammal groups, Fernando and Melnick^24^ first suggested the use of the sex-specific variants of the Zinc-Finger gene within elephants (herein referred to as ZFX and ZFY). They promoted a direct analysis of potential “double-peaks” in chromatograms to establish the genotype, but this approach has received little audience. Instead, genotyping the Zinc-Finger alleles has long relied upon agarose electrophoresis of restriction fragments length polymorphism (RFLP) using a differential BamHI digestion^2,24^. The original ZFX/Y assay for the elephants required the amplification of a 450 base pair (bp) long amplicon. Two studies independently suggested reducing the amplicon length to improve the success rate for potentially degraded non-invasive samples: Chakraborty et al.^25^ developed a 265 bp long assay in addition to a Y-specific internal primer to segregate both sexes directly at the PCR stage, whereas Munshi-South et al.^26^ shortened the PCR design down to 150 bp while retaining the RFLP approach.

Due to potential contamination risks related to the high level of conservation of the Zinc-Finger gene among mammals, some authors have developed another type of assays^27,28^. In this second strategy, the sexing relies upon the amplification of sex-specific amplicons, mostly the SRY gene present only on the Y chromosome. The risk of false-negative results (i.e. excess of female genotypes) is very high as many factors but the sex might cause a non-amplification of the SRY fragment: PCR inhibitors, primer mismatches, insensitive PCR conditions, etc. Gupta et al.^27^ attempted to circumvent that limitation by complementing their SRY test with a separate, non-sex specific, amplification of a mitochondrial fragment. However, in the absence of any reciprocal sensitivity analysis of the SRY and mitochondrial assays, the risk that false female signatures be recovered remains: the sole positive amplification of the mitochondrial fragment may reflect its much higher copy number in the cell than the nuclear Y chromosome. More recently, Ahlering et al.^28^ proposed a multiplex PCR approach of three sex-specific nuclear markers: SRY and AMELY2 on the Y, and PLP1 on the X. Their amplicon lengths were below 200 bp, with the longest for the X marker. This design mitigated the risk of false-negative assignation to the female sex: in case all three amplicons were negative, no sex was inferred. Mondol et al.^12^ refined that multiplex methodology in order to automatize the genotyping via the fluorescent tagging of amplicons from almost one thousand ivory seizures. Their overall success rate of 65.5% was positively correlated with the observed length of amplifiable DNA in the samples. This outcome exemplifies that, despite the amplicons being relatively short (between 71 and 192 bp), over a third of the specimens could not be sexed due to the DNA degradation in the samples.

The published material from both elephants and mammoths leads to the invariant observation that a simple, cost-effective, and yet sensitive method to derive the genetic sex of elephantine specimens is pending. To limit the risk of false-negative assignation while using a single assay, we have preferred to build upon the Zinc-Finger genotypes with two allelic copies present in both sexes^24^. Here we propose to re-invent the Zinc-Finger sexing assay to meet three key criteria. (I) The novel assay needs to be as short as possible to be usable on highly degraded DNA sources—whether modern or ancient—while maintaining sufficient sex-specific variation to discriminate ZFX and ZFY alleles unambiguously. (II) The PCR primers must be equally efficient for both alleles across all elephantine taxa to exclude any potential bias, and yet minimize the risk of contamination, particularly with humans. (III) The sexing assay should discriminate both ZFX and ZFY alleles at the real-time PCR stage, without the need for any other equipment or experiment downstream the amplification step. We assess the performance of this novel assay in three different experimental contexts: with an in vitro analysis of standard quantitative series of both ZFX and ZFY elephantine alleles, and through two case studies that involve DNA extracts from elephant feces from Uganda as well as woolly mammoth bones from Siberia.

## Materials and methods

### Design of the novel Zinc-Finger TaqMan assay

**Figure 1:**
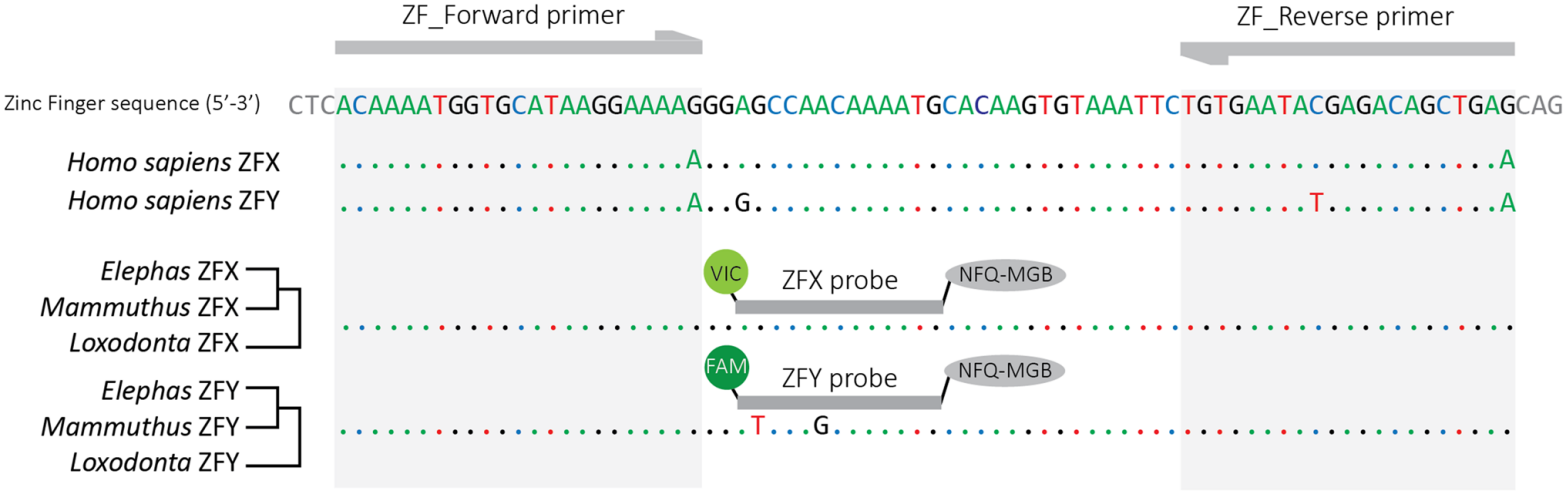
Alignment of the Zinc-Finger amplicon of interest for the ZFX and ZFY alleles from humans and elephantine taxa: *Loxodonta* (African elephants), *Elephas* (Asian elephants) and *Mammuthus* (mammoths). The top sequence represents the elephant ZFX allele; identities are indicated by dots. Primers and MGB probes are displayed in annealing position.

In order to establish the level of sequence conservation of the Zinc-Finger gene within the elephantine taxa, we aligned the known ZFX/Y regions for both alleles and each genus using Geneious R9. For the Asian elephants (*Elephas maximus*), we used the previously published Sanger sequences^24^ (Supplementary Table S1). For the woolly mammoths (*Mammuthus primigenius*) and the African elephants (*Loxodonta africana and Loxodonta cyclotis*), due to the lack of actual Zinc-Finger sequences deposited in sequence databanks, we recovered the corresponding sequences via the mapping of published whole-genome NGS reads from known male specimens^20,29^ (Supplementary Table S2). This alignment shows the complete conservation of the signatures discriminating the ZFX and ZFY alleles in Asian elephants at the scale of the elephantine subfamily. Low coverage data of this region (4X) are also available for the American mastodon (*Mammut americanum*; Supplementary Table S2), an extinct proboscidean species, which is a quite distant relative to the elephantine taxa: their most recent common ancestor dates back to 25–30 Mya (clade Elephantimorpha^30^). The comparison with the elephantine sequences strongly suggests the antiquity of these allelic signatures within the proboscideans (Supplementary Fig. S1). Conversely, when added to our comparison, the overlapping ZFX/Y sequences of modern humans show several fixed divergent positions from the elephantids (Fig. 1).

We designed one pair of primers: ZF_Forward (5′-ACAAAATGGTGCATAAGGAAAAG-3′; Tm = 58.9 °C) and ZF_Reverse (5′-CTCAGCTGTCTCGTATTCACA-3′; Tm = 60.3 °C), which promote the amplification of a 74 bp long amplicon surrounding two sex-specific polymorphic sites. We chose priming sites exhibiting fixed divergent positions with human ZFX/Y sequences—specifically the final 3′ position of the forward primer—to reduce the risk of amplification of human contaminants. Based on the melting temperatures of the chosen primers, we designed two sex-specific Minor Groove Binding (MGB) fluorescent probes diverging from each other by two of their 13 nucleotides (Fig. 1): ZFX 5′-VIC/AGCCAACAAAATG/NFQ/MGB-3′ (Tm = 69.0 °C) and ZFY 5′-FAM/ATCCAGCAAAATG/NFQ/MGB-3′ (Tm = 68.8 °C), labelled with the two fluorescent dyes used by default in bi-allelic discrimination^31^, and manufactured by Applied Biosystems (Foster City, CA).

### In vitro sensitivity experiments

To address the sensitivity of our assay, we first generated sex-specific quantitative standards: we diluted a male mammoth DNA extract (Lyakhov mammoth; Supplementary Table S4) until the point when real-time PCR reactions using this dilution as a template would only yield the amplification of one or the other sex-specific allele (or no product at all). We pooled three reactions for which only the X allele was detected in one microtube, and three other Y-positive reactions in another microtube. Each pool was purified using the minelute PCR purification kit (Qiagen, Venlo, NL) and concentrated separately in 10 µl of EBT buffer (Qiagen EB buffer supplemented with 0.05% Tween-20). We quantitated each sex-specific standard using the Qubit High Sensitivity assay kit (Invitrogen, Waltham, MA) and prepared a tenfold dilution series ranging from 10^10^ copies down to 10^−1^ copy per µl. Standard series were stored in frozen aliquots and thawed only before use.

We analyzed the sensitivity of the assay in two dimensions: (I) the sensitivity of the PCR amplification in absolute copy numbers and (II) the relative sensitivity of both X- and Y-specific allele diagnostics. We first tested the general sensitivity of the assay using a SYBR Green I approach, with 1X Sso-Advanced Supermix (Bio-Rad, Ipswich, MA) and a standard series of each allele (10^5^ down to 10^−1^ each), using 6 replicates of the standards at the low end (2 × 10^0^ and 2 × 10^−1^). We then evaluated the reciprocal sensitivity of each MGB probe via a TaqMan reaction using a standard series ranging from 10^5^ down to 10^0^ each, with three replicates for the latest. For probe-based PCR reactions, we used the dedicated TaqMan Fast Advanced Master Mix (Applied Biosystems, Foster City, CA) which contains dUTP and Uracyl-N-glycosylase (UNG) pre-treatment steps to avoid PCR contamination from carryover PCR products.

### Quantitative PCR optimization and genotype analyses

We compared the behaviour of TaqMan reactions with various combinations of primer concentrations between 400 and 900 nM, final probe concentrations ranging between 200 and 600 nM, and an annealing/extension temperature gradient (55–65 °C). The best sensitivity was obtained around 60.5 °C regardless of the reagent concentrations: the Cq of the standards were retarded by up to 0.8 or 1.2 cycles when lower or higher temperatures were picked, respectively. Balanced MGB probe concentrations systematically yielded a higher response of the FAM probe over the VIC one (up to 150%), and sometimes caused a shallow crosstalk-signal artifact within the VIC detection range. Implementing uneven probe concentrations—increasing VIC by one-third and lowering FAM by as much—addressed both issues. We thus adopted the following conditions for all subsequent experiments: final reaction volumes of 15 µl with 1X of TaqMan Supermix, 800 nM of each primer, 375 nM of Y-FAM probe, 525 nM of X-VIC probe, and 1–2 µl of DNA extract.

We performed all PCR reactions on a CFX-96 real-time thermocycler (Bio-Rad, Ipswich, MA) using the following 2-step conditions: after a first denaturation of 2′ at 95 °C, we performed 40 cycles of 95.0 °C 10 s and 60.5 °C 35 s. We conducted the allelic discrimination from the qPCR output with the CFX-Manager software v3.1 (Bio-Rad, Ipswich, MA) using the following set of parameters: baseline subtracted curve fit, quantification cycle (Cq) determined via a single threshold set to 10% of average plateau fluorescence (measured in Rescaled Fluorescence Units, RFU), call of alleles on the last PCR cycle.

### Specificity analyses

We investigated the level of specificity of our assay against human contaminants via straight qPCR attempts with various concentrations of control human genomic DNA (Thermofisher, cat. number 4312660): 1, 5, and 25 ng per reaction. We complemented this analysis with an in silico assessment of our assay: we used BLASTn^32^ to analyze the ‘nr’ collection database in GenBank, and identify which taxa shared sequence identity with at least one of our primers. Among those hits, we focused on the putative sympatric taxa of elephantids (modern and extinct) for which we aligned the available ZFX/Y fragments.

Although the risk of non-specific detection is extremely low with an MGB-TaqMan methodology^31^, we chose to monitor the specificity of PCR design in our case study experiments. We prepared two pools—one per case study—from all positive PCR reactions from actual specimens across an entire replicate series. We transformed these pools in double-indexed Illumina libraries^33^ and performed a shallow sequencing of each (in paired-end 2 * 75 bp).

### Case study on elephant fecal extracts

We conducted the fecal sampling of wild elephants from November 2016 to January 2019 in Sebitoli area in the vicinity of Kibale National Park (south-western Uganda). The wildlife of this forest area, located at the north of the protected area, is studied by the Sebitoli Chimpanzee Project/Great Apes Conservation Project and the Muséum national d’Histoire naturelle (MNHN, Paris, France). Commercially logged in the 1970s, the Sebitoli forest is now composed of 70% of regenerating forests and only 14% of old-growth forest^34^. In areas adjacent to Kibale, human population density is high^35^ (circa 300 inhabitants/km^2^). They grow monocultures such as tea fields, eucalyptus, and banana plantations as well as crops like maize, which attract elephants and primates out of the forest. This survey is part of a project aiming at mitigating the human-wildlife conflict at the edge of the protected area in the framework of the Memorandum of Understanding SJ 445-12 between MNHN, Uganda Wildlife Authority, and Makerere University in Uganda and the MoU between UWA and GACP.

To avoid the repeated sampling of the same individuals, we collected only once when we encountered several dung boli of similar size on the same day and location. Since female elephants live in close family groups^36,37^—while the adult males are mostly solitary—this strategy made the sampling of male dung more likely than female ones. A quantity of 10 to 15 g of feces was stored in 70% ethanol for 24 h. After removing the supernatant, feces were placed in gauze on silica gel beads and stored at ambient temperature until processed in the laboratory. After removing the largest vegetal compounds, between 150 and 200 mg of dried feces were extracted with the Power fecal DNA Isolation Kit (MoBio, Carlsbad, CA). The DNA extraction was performed in France, at the modern lab of the ‘Plateau de Paléogénomique et Génétique Moléculaire’ (P2GM platform) from the MNHN. Total DNA yields from the extracts, as measured with a NanoDrop 2000 (ThermoFisher Scientific, Waltham, MA), ranged from 2.9 to 186.7 ng/μl (Supplementary Table S3).

To validate the assay, we used a set of 12 elephant extracts for which sex was known a priori: six male and six female specimens. We then implemented our assay in a case study that involved 91 specimens of unknown sex. Two PCR replicates per individual extract were performed, in parallel with a total of 7 PCR negative controls (NTC for ‘No Template Control’ reactions).

### Case study on mammoth ancient DNA

Over the last 20 years, we have gathered several dozens of woolly mammoth samples that have been used in various paleogenetic analyses^38,39^. They are part of a broad comparative genomics project of diachronic specimens from Beringia which objective is to address the diversity and gene flow throughout the Late Pleistocene populations of woolly mammoths. Here we attempted to derive the genetic sex for a subset of 29 specimens using the novel assay. These samples all come from the Late Pleistocene in Siberia, and the radiocarbon-dated specimens range from 4420 up to beyond 50 ky BP (Supplementary Table S4).

DNA extractions and PCR setup of mammoth samples took place in the dedicated ‘ancient DNA cleanroom’ at the P2GM platform, which is physically isolated from the modern lab. We used a protocol previously published for DNA extraction from bone^39^ and extracted the specimens in 5 different series—each along with one extraction blank. We first tested six specimens of known sex (thanks to a morphological diagnosis): Lyakhov, Jarkov and Oymiakon (all males), 2001/174, Lyuba and Khroma (all females). We then implemented the assay on 23 extracts of unknown sex together with each extraction blank, several NTCs, and one absolute standard series to establish the number of template molecules for each X and Y allele available from our mammoth extracts.

Our sexing assay relies on the identification of one homozygous genotype (female) and one heterozygous genotype (male) via a bi-allelic target. In such a design, the risk of false assignation of a male to the female genotype due to allelic dropout of the Y allele is a limitation, particularly when working with templates of low DNA content^40,41^. We carried out all mammoth PCR reactions in triplicates, to comply with the multi-tube strategy developed to control for that risk. The implementation of a quantitative PCR framework in our sexing assay provided us with the ability to refine the estimate of the accuracy of the genotypes. Taberlet et al.^41^ showed that (i) the allele amplification of a bi-allelic marker behaves stochastically for very dilute samples, and (ii) for a known amount (U) of diploid genome copies in a reaction, the probability of allelic dropout can be precisely modeled (Supplementary Fig. S4). We posited that the sum of ZFX and ZFY allele copy numbers per reaction inferred via qPCR is a relevant proxy of this amount among our samples—a reasonable assumption when one considers that Zinc-Finger is a single copy nuclear gene. We derived the absolute copy number (CN) based on the Cq calculations for both Y-FAM and X-VIC between the positive specimens and the corresponding standard series. We then used this metric to estimate the probability P_XX_ of allele dropout per reaction for a true heterozygote, based on Taberlet et al.’s model. For each specimen, the theoretical risk of wrongly being deemed a female due to allelic dropout thus translates as (P_XX_)^n^ from the binomial distribution of parameters n and P_XX_, where n refers to the number of PCR attempts that yielded a genotype (Supplementary Table S6).

## Results

### Sensitivity of the assay

**Figure 2:**
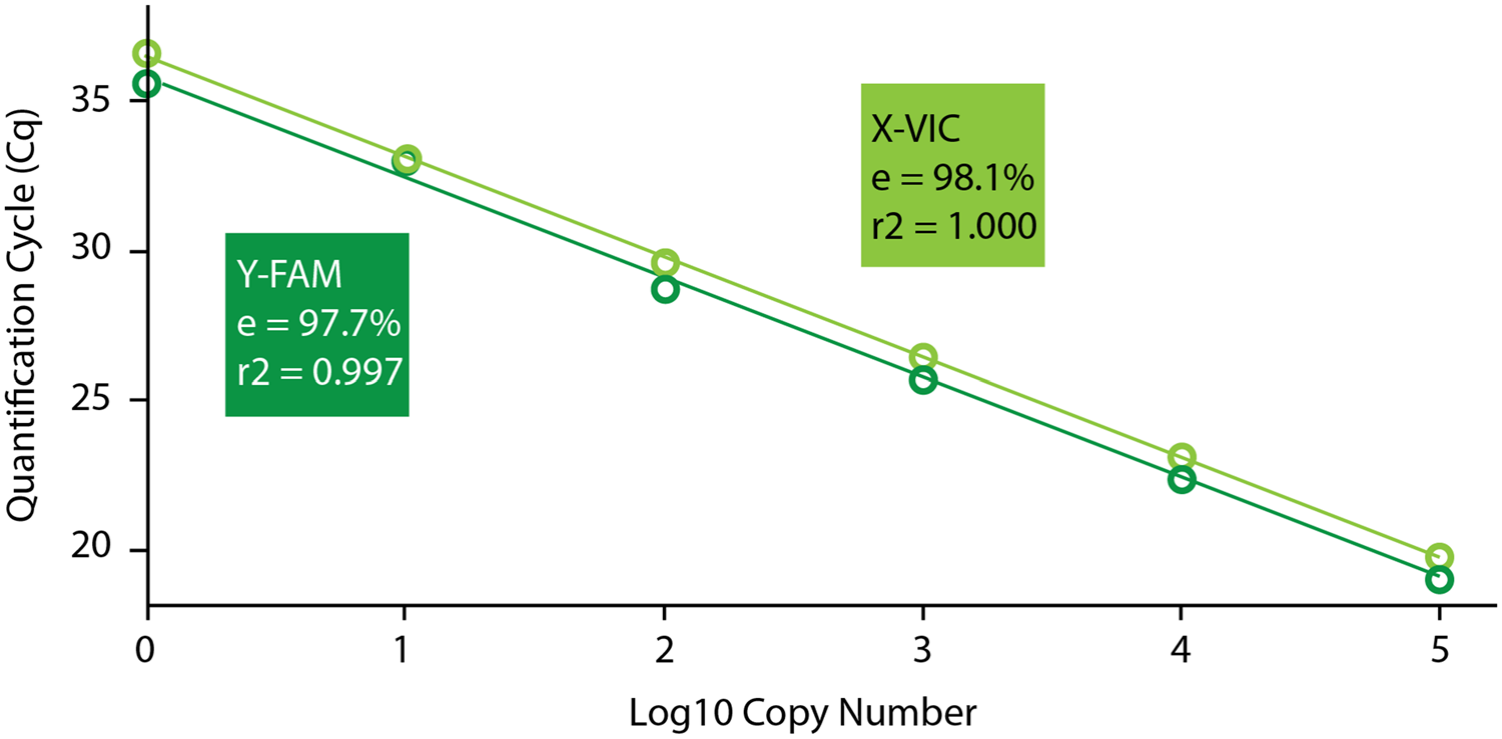
Standard curves for each sex-specific Zinc-Finger allele. Amplification efficiency (e) and correlation factors of the standard series (r2) are indicated for each fluorescent probe.

For the standard series analysis based on SYBR Green quantitation, all PCR reactions were positive when we used at least two template molecule copies (Supplementary Table S5). Conversely, out of six reactions using 0.2 template copies, only one came up with a positive amplification. When the ZFX/Y alleles were assessed separately via TaqMan quantitation, all reactions were positive down to one copy of each allele, but not both alleles were detected for the three replicates performed at that concentration: one replicate detected only the X allele, one only the Y allele, and the last one detected both. The examination of the standard curve for each sex-specific allele taken independently shows very similar kinetics between the probes (Figs. 2, S3): their measured efficiency is approximately 98% for both, with a slight delay in the X-VIC response of about a half cycle throughout the reaction. For each allele, the standard curve shows a linear quantitative response (once log-transformed) from 10^5^ down to one copy with a correlation factor r2 > 0.997.

### Specificity analyses

The experimental tests using human DNA as qPCR templates did not yield any detectable amplification product, even with the highest amount used (25 ng). In parallel, we sequenced almost 400 k reads based on our pooled positive PCR products from our elephant and mammoth datasets. Over 99.5% of the qualified reads could be unambiguously mapped to our target fragment within the elephantine Zinc Finger Gene, whereas the remaining reads were inconclusive due to their short length.

The in silico similarity analysis of the primers revealed that no documented species outside the elephantine group shares a perfect match with both primer sequences. The closest matches for the primers are found among three mammal groups: some other afrotheres than the proboscideans, some primates, and some carnivores (Supplementary Fig. S2). The last 3′ position of the ZF_forward primer is a ‘G’, fixed for both ZFX and ZFY alleles within all elephantine taxa, whereas it is almost always an ‘A’ in other mammals, like in humans. Some rare exceptions are documented for potentially sympatric species where one Zinc-Finger allele displays an identical ZF_forward sequence to the elephantine sequences. They concern the chimpanzees’ ZFX (which differ from all other Great Apes in that regard) and some canids ZFY. Nonetheless, the canids’ ZFY always possess at least one fixed variant from the elephants’ alleles in our probe range, which should preclude its fluorescent detection.

### Cases studies

**Figure 3:**
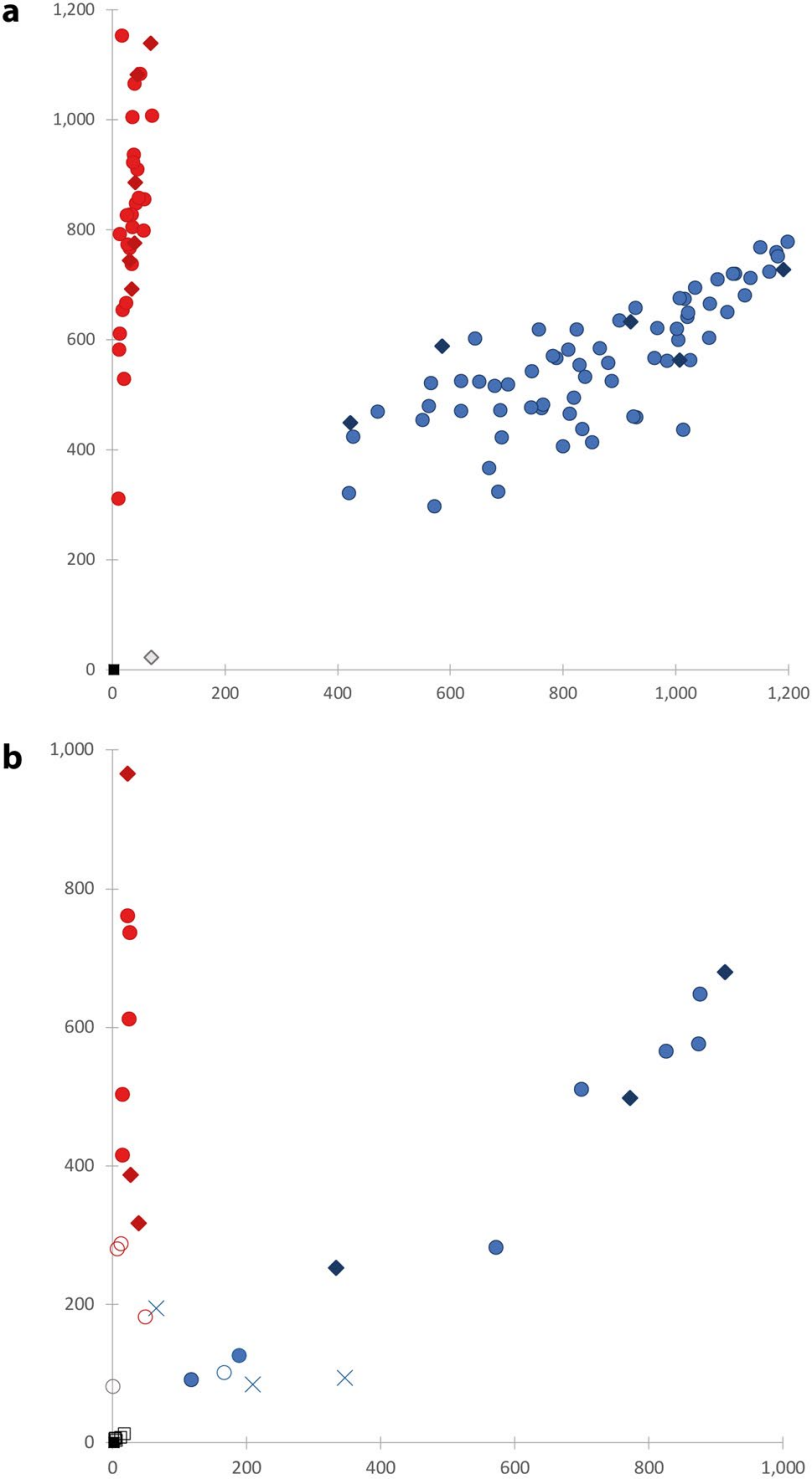
Allelic discrimination plots for the two case studies. Axes values in Rescaled Fluorescence Units (RFU). Male genotype calls are displayed in blue and female in red (supported calls are filled, putative calls are empty). Filled diamond: specimens of known sex a priori; filled circle: specimens of unknown sex a priori; ×: specimens with inconsistent calls; filled square: NTC; open square: extraction blanks. (**a**) Elephant case study. (**b**) Mammoth case study.

Out of the 12 elephant test samples, all but one—which exhibits the lowest total DNA concentration in our set—provided positive amplification products. The genotype was accurately called twice for the 11 positive extracts: five males out of five and six females out of six (Supplementary Table S3). For the batch of elephants of unknown sex, all 91 specimens provided a molecular sex, while all the NTC remained negative (Fig. 3a). Thus, the call rate of the assay for all tested elephants was > 99% (204/206). Due to the absence of normalization in DNA amounts prior to the test^42^, we observed an extended trail of plots for the homozygous genotype and a wide cluster spread for the heterozygous genotype. Yet, the genotyping of both molecular sexes was straightforward and both replicates consistently yielded the same genotype for each specimen: 32 XX (females) showed no amplification of the Y-FAM allele (no Cq and RFU < 100 for Y-FAM) whereas 70 XY (males) had Cq for both alleles. We observed no homozygous YY genotype (i.e. no Cq and RFU close to 0 for X-VIC only).

**Figure 4:**
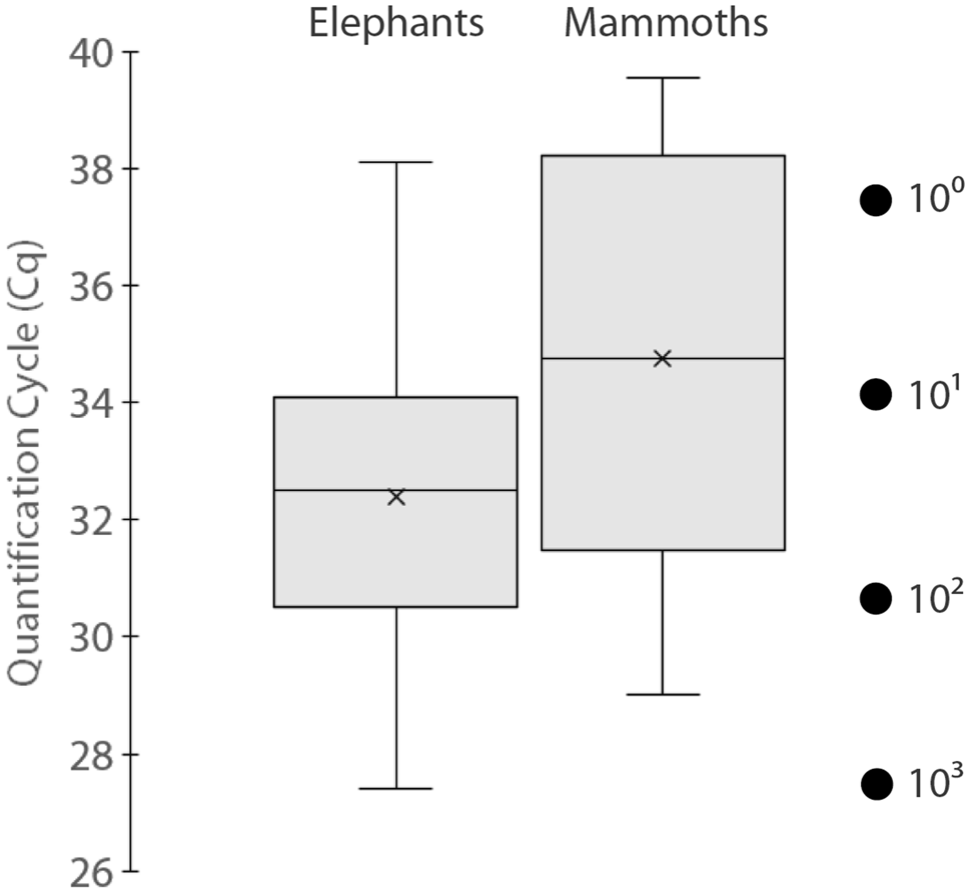
Boxplot distribution of the quantification cycle (Cq) range for the X-VIC allele (detected in both sexes). Left: modern elephant dung samples (n = 105); right: ancient mammoth bone samples (n = 26). Filled circle at the far right indicate the Cq of the quantification standards for comparison.

For the mammoth case study, three specimens remained negative for all three replicates (both RFU close to 0, like the NTCs and extraction blanks), and four others amplified only twice. Among the 74 positive PCR replicates, four yielded non-discriminant final RFU values, which led to inconclusive calls—hence an actual positive call rate of 70/87 reactions (80%). The genotype calls are distributed as follows: 32 XX, 35 XY, and three YY (Table 1). Out of the 24 extracts that provided a putative genotype from at least two independent PCR reactions, 21 consistently yielded the same genotype across positive replicates: 11 females (XX) and 10 males (XY). Both male and female specimens of known sex a priori yielded results strictly congruent with the expected genotype (Table 1). However, a fraction of the ancient samples yielded low fluorescence values for both probes (i.e. generally below 200 RFU). In parallel, their Cq were generally higher than those obtained for the elephant dung, with more than half of them beyond the 35th cycle (Fig. 4). When translated into copy numbers, the average CN estimates per individual reaction cover three orders of magnitude: one half of the positive samples had beyond 20 Zinc-Finger copies whereas the other half never exceeded 10.3 copies per reaction. The consistency of the genotype calls per reaction was highly correlated with the CN value: the specimens for which PCR replicates provided inconsistent results all rank in the last ten samples when ordered by descending CN average (Table 1).

**Table 1:** Genotyping reactions summary and inferred sex for the 26 mammoth specimens that yielded positive amplifications.

Specimen	Replicate details					Average RFU		Average CN			Inferred sex	
ID	PCR+	XX	XY	YY	?	Y-FAM	X-VIC	Y-FAM	X-VIC	Total		(P_XX_)^n^
Khroma (F)	3	3				23	966	0.0	887.5	887.5	Female	< 0.1%
2005/931	3	3				23	760	0.0	678.3	678.3	Female	< 0.1%
2005/918	3	3				26	736	0.0	500.3	500.3	Female	< 0.1%
2005/898	3		3			826	565	237.2	214.8	452.0	Male	< 0.1%
Oymiakon (M)	3		3			772	498	174.5	229.9	404.4	Male	< 0.1%
2005/897	3		3			874	575	143.7	135.4	279.1	Male	< 0.1%
Lyakhov (M)	3		3			913	680	121.0	151.8	272.8	Male	< 0.1%
2005/915	3		3			700	510	81.0	86.3	167.3	Male	< 0.1%
2005/924	3		3			877	647	50.8	34.9	85.7	Male	< 0.1%
WR2	3	3				25	611	0.0	53.3	53.3	Female	< 0.1%
2000/174 (F)	3	3				27	387	0.0	24.8	24.8	Female	< 0.1%
2005/999	3	3				15	503	0.0	22.1	22.1	Female	< 0.1%
2005/913	3		3			572	282	13.6	7.1	20.7	Male	< 0.1%
2005/900	3	3				16	415	0.0	10.3	10.3	Female	< 0.1%
Jarkov (M)	3		3			333	253	3.0	3.7	6.7	Male	< 0.1%
Lyuba (F)	3	3				39	317	0.0	3.3	3.3	Female	0.4%
2005/945	3		2	1		346	95	2.1	0.7	2.8	Male	0.7%
2001/451	2	2				13	287	0.0	2.3	2.3	Female ?	5.1%
2002/489	3		3			189	126	0.9	0.9	1.8	Male	1.9%
2003/838	2			1	1	167	101	0.6	1.0	1.6	Male ?	28.7%
2005/927	3	1	2			65	195	0.2	1.4	1.6	Male	2.4%
2000/165	3	2			1	50	181	0.3	1.2	1.5	Female ?	8.8%
2000/187	3	1	1	1		209	85	0.8	1.7	1.5	Male	2.6%
2005/904	3		3			118	91	0.7	0.7	1.4	Male	2.9%
2000/175	2	2				8	280	0.0	1.2	1.2	Female ?	10.9%
2000/176	2				2	1	81	0.0	0.6	0.6	?	na

The specimens are presented by descending total copy number (CN) per reaction. When the sex of the specimen was known a priori, it is indicated next to its ID in parentheses. Replicate details provide the number of positive reactions (PCR+) broken down by genotype (homozygous XX or YY, heterozygous XY, and inconclusive ‘?’). Average fluorescence (RFU, given in arbitrary units) and copy numbers per reaction (‘total’ being the sum of the Y-FAM and X-VIC allele counts) are provided. The (P_XX_)^n^ column refers to the theoretical risk of allelic dropout for each specimen (see main text and Supplementary Table S6 for details).

## Discussion

When we started this study, our primary objective was to promote a novel molecular sexing assay that would work for all elephantine taxa (extinct mammoths and modern elephants), in the context of degraded DNA material. To be a useful diagnostic tool, this assay needed to be easy to set up and rapid to perform, require the minimal possible equipment and yet remain economically relevant. To reach those objectives, we adopted the TaqMan-MGB fluorescence approach, which has been the leading technique for targeted SNP detection in the range of PCR allelic discrimination methods over the last two decades^31,43,44^. Although it relies on SNP signatures on the sex-specific alleles of the Zinc-Finger gene that have been known for 20 years, our assay represents the first implementation of this methodology for the determination of the sex of elephantine taxa.

The use of the minor-binding-groove probe technology allowed us to design a very short assay (74 bp) to accommodate degraded DNA sources. It is six times shorter than the original Zinc-Finger assay^24^ and still twice shorter than the construct proposed by Munshi-South et al.^26^. Previous quantitative work on ancient DNA has shown that a twofold increase in amplicon length might lead to a tenfold decrease in copy number^22^. We thus expected that our length adjustment should extend the range of usable (degraded) samples to determine the sex, compared with previously published sexing assays for elephants. The very high level of sensitivity that the novel assay exhibits also warrants its usability in that regard. For the selected set of experimental parameters and reaction conditions, we have shown that it is sensitive down to two template copies per reaction—whereas no such appraisal of the sensitivity is available for the previously published assays. Furthermore, our analysis of the ZFX and ZFY allele standard series revealed that it is similarly sensitive and efficient for each allele (Fig. 2). Despite a visual delay of approximately a half cycle in the X-VIC response compared with the Y-FAM signal, the difference is merely imputable to the lower fluorescence level of the VIC probe.

Despite its short length, our assay is also very specific, thanks to the dual hybridization of specific PCR primers and allele-specific probes (Fig. 1). Firstly, the observed specificity of each probe towards its target allele is total: no false detection was caused by crosstalk. Secondly, the clonal parallel sequencing of over 120 PCR products of our elephants and mammoths samples has confirmed the specificity of the amplification towards the Zinc-Finger gene even when using low endogenous DNA content like in elephant fecal samples. Thirdly, the assay has the advantage over the formerly published Zinc-Finger RFLPs to minimize the risk of contamination from other taxa, a concern initially reported by Ahlering et al.^28^. Thanks to our design of the ZF_forward primer—with a 3′ position distinctive of the elephantine taxa against a vast majority of known mammal sequences (including humans)—the likelihood to amplify any product from contaminant DNA is greatly reduced. Our in vitro tests using human DNA thus demonstrated that the amplification of human DNA is excluded, even in concentration levels higher than might be expected from secondary contamination (on the field or in the lab).

Some extra benefits in the use of a pure qPCR approach for the molecular detection of sex arise from its simplicity^31^. It is very time and labor-effective because the qPCR reaction is the only experimental step to perform after extraction so that the genotyping of hundreds of DNA extracts can be performed in less than 2 h. There is no need for electrophoresis equipment or consumables. On economic grounds, despite the cost related to the original acquisition of the necessary real-time thermocycler and fluorescent probes (that are worth hundreds of reactions), the assay remains inexpensive. Thanks to our custom reaction adjustments, the price per reaction was below 1.5 dollars in our experiments.

The comparison of the outcome for our two case studies is very informative. In both cases, the assay exhibits a high call rate for actual samples: > 99% observed in modern elephants and 80% in mammoths, while remaining negative for all negative controls. It is noteworthy that all 40 positive PCR reactions for specimens of known sex a priori provided the accurate genotype—twenty-two from elephants and 18 from mammoths. This outcome allows us to calculate that the accuracy of our assay is at least > 92% (with a *p* value of 0.05). These results confirm the usefulness of this approach to determine the sex from degraded DNA material like dung, bone or ivory, for modern elephants as well as for ancient remains. Although we did not test the assay for a set of Asian elephants, the published knowledge concerning the diversity of their ZFX/Y alleles^2,24^ as well as our alignments of the ZFX/Y alleles within available proboscidean taxa (Fig. 1) advocate for its relevance in that lineage too.

Despite these similarities, it is in their differences that these experiments are the most enlightening. The frequency of positive genotype calls is not only lower in the ancient samples, but it is also less reproducible: while the results of the replicates series were 100% congruent for the 106 positive elephants, six mammoths out of 26 (23%) yielded inconsistent or inconclusive genotype calls during the replication process. Unsurprisingly, the reason for this discrepancy lies in the observation that the mammoth sample set is generally more degraded than its elephant counterpart, as revealed by their Cq distribution (Fig. 4). Among the mammoth extracts themselves, the decrease in amplification success rate—as well as the inconsistency in genotype call—is only observed for the 10 mammoths with the lowest CN (i.e. less than 3 copies per reaction; see Table 1). With such low DNA templates, the stochasticity of the PCR amplification process becomes a pervasive issue due to allelic dropout, and the selection of a multi-tube strategy—three replicates per sample—is a method of choice to evaluate the accuracy of the genotypes^40,41^.

Although the determination of the sex might seem straightforward when all three replicates exhibit the same genotype, the situation needs to be addressed separately for putative heterozygous (male) and homozygous (female) specimens. The three independent occurrences of the XY genotyping for 10 specimens warrant their male status with a high level of confidence^41^. Aside from these univocal results, four other putative male specimens deserve some attention. It is noteworthy that three PCR replicates provided a homozygote YY genotype for three different samples. This spurious genotype suggests the allelic dropout of the X allele from male samples. For the specimen 2003/878, this YY genotype was obtained for the sole discriminant PCR reaction, so that its robust sex determination is pending—one cannot exclude random contamination to explain this result. For the two other specimens concerned, however, the hypothesis of a male genotype is well supported: 2005/945 was typed as XY for the other two replicates, and 2000/187 was typed once XY and once XX in the other two replicates. The latter specimen exemplifies the situation where a stochastic dropout of either the X or the Y happened in two out of three replicates. One final putative male specimen deserves some attention: 2005/927 yielded two XY replicates and a single XX replicate, which is the symmetrical situation to specimen 2005/945, and can be explained by the allelic dropout of the Y allele in one replicate. These outcomes highlight the importance of the replication process, which allowed us to detect that the two specimens 2005/187 and 2005/927 are males, whereas a single PCR reaction could have led to the opposite conclusion.

As discussed above, three positive replicates are usually sufficient to identify a male specimen, despite allelic dropout. However, the reciprocal confirmation of a female genotype is somewhat trickier: up to 7 positive replicates might be necessary to reach a 99% confidence in the assignment of a homozygous genotype^40,41^. Because of the rarity of the mammoth DNA, we could not spare more than three reactions per specimen. Instead, we made use of our CN estimates to infer the sample-specific risk of allelic dropout per reaction. Associated with the number of positive homozygous replicates, it allows deriving the probability of a false homozygous genotype due to allelic dropout. When integrating the CN in our calculations, the probability of error was < 1% for the 8 putative female specimens which yielded 3 XX genotypes, confirming those hypotheses. For the remaining three specimens: 2001/451, 2000/165, and 2000/175, the three replicates yielded only two XX genotypes and one negative or inconclusive reaction. The availability of only two positive genotypes affects the accuracy of the genotyping negatively, and the risk that these specimens are genotyped as females while being actually males rises to 5.1, 8.8, and 10.9% respectively. Although the female sex is the most likely for these three specimens, they all require at least one extra positive XX replicate to reach a confidence level > 99%.

No targeted-PCR approach pre-existed to determine the sex of such ancient remains and, in fact, very few nuclear sequences of mammoths were published until the advent of shotgun sequencing of NGS libraries^45,46^. This difficulty is exacerbated by the differential, in genomic copy number, between the mitochondrial and the nuclear genomes: previous work has shown that it is generally between two and four orders of magnitude in favor of the mitochondrial genome^22^. Most of the published PCR assays are thus using mitochondrial DNA and are only of use for taxonomic or phylogeographic assignation^47,48^. The latest of the mitochondrial assays aiming at identifying the taxon (elephant or mammoth) from ivory DNA obtained a success rate in PCR as high as 96.7% from ancient tusk^15^. In comparison, our rate of genotype call of 80% appears very promising. In conjunction with a multi-tube approach, it allowed us to determine the sex with a confidence level of at least 99% for 19 specimens—eight females and eleven males—out of 29 mammoths (65%). The genotype of three extra putative females and one putative male requires additional replicates for confirmation. This rate of accurate sexing from mammoth specimens is lower than the ones published using the screening of NGS libraries^17,23^ (> 90%), but it comes at a fraction of the cost and labour, and we believe both methodologies are complementary.

On a side note, we’d like to emphasize how the number of PCR cycles is important to monitor the risk of false genotype assignation in an assay such as ours. With as few as 40 cycles as in our experiments, low CN samples simply don’t reach the plateau phase of the PCR and yield low final RFU values which allow to discriminate them easily from the samples with higher CN. We thus discourage implementing higher numbers of PCR cycles for this assay for they would blur the difference in RFU values between samples and could lead to a spurious high ZFX RFU only from low CN male specimens that would be hard to detect without an absolute quantification. Furthermore, we suggest a standard series be implemented when late Cq prevail (hence low CN) in your samples: the combined use of CN and final RFU values allows to discriminate the genotype calls by sex and by accuracy.

We produced in vitro analyses of a novel TaqMan-MGB assay designed to determine the sex of elephantine specimens in the context of degraded DNA samples. They confirmed that it meets our expectations in terms of (I) efficiency of the molecular diagnostic, (II) an unrivaled level of sensitivity even for low amounts of degraded DNA, and (III) an affordable, rapid, and easy-to-implement, experimental framework. In practice, it is of course useful only for the biological samples from which at least a few nuclear copies of the target are present per reaction, and one should remain cautious when dealing with such low DNA content samples for which multiple replicates are mandatory to support the genotype call. With the in vivo procedure adopted in this study, we could confidently determine the sex for all the elephant specimens but one, and for more than a half of the woolly mammoths analyzed, which is unprecedented via nuclear targeted PCR. We believe these results advocate for the usefulness of this method to determine the sex of a wide range of degraded samples from modern or extinct elephantine taxa: it thus should prove useful to the population geneticist, the conservation biologist but also the zooarchaeologist and the paleogeneticist.

## Supplementary Information

Supplementary Informations.

## Data Availability

The sequence reads produced for this manuscript are publicly available at ENA with run accession numbers ERR5059481 and ERR5059482. The published sequence data analyzed in this manuscript are listed in Supplementary Tables 3 and 4.
