# Supplementary material for: Molecular sexing of degraded DNA from elephants and mammoths: a genotyping assay relevant both to conservation biology and to paleogenetics
Source: Sci Rep. 2021 Mar 31;11:7227. doi: 10.1038/s41598-021-86010-x (PMC8012363; doi:10.1038/s41598-021-86010-x)

**Supplementary Information**

**Molecular sexing of degraded DNA from elephants and mammoths: a genotyping assay relevant both to conservation biology and to paleogenetics**

Laetitia Aznar-Cormano, Julie Bonnald, Sabrina Krief, Nelson Guma, Régis Debruyne

| SUPPLEMENTARY information | pages |
| --- | --- |
| Table S1 | 2 |
| Table S2 | 2 |
| table S3 | 3-7 |
| Table S4 | 8-10 |
| Table S5 | 11 |
| Table S6 | 11 |
| Figure S1 | 12 |
| Figure S2 | 13 |
| Figure S3 | 14 |
| Figure S4 | 14 |

# Supplementary Tables

### Table S1: Sanger sequence datasets used in the Zinc-finger alignments

| Taxon | Genbank accession | Reference | Allele |
| --- | --- | --- | --- |
| *Elephas maximus* | AF393751 | Fernando & Melnick 2001 | ZFX |
| *Elephas maximus* | AF393752 | Fernando & Melnick 2001 | ZFY |
| *Homo sapiens* | NM002410 | Saito et al. 1994 | ZFX |
| *Homo sapiens* | NM002411 | Saito et al. 1994 | ZFY |

### Table S2: Published Illumina sequence datasets used in the Zinc-finger alignments

| Taxon | Dataset reference | reference | Specimen info | Number of reads | N Aligned reads to the Zinc-Finger amplicon | N diagnostic reads ZFX/ZFY |
| --- | --- | --- | --- | --- | --- | --- |
| *Mammuthus primigenius* | ERR852028 | Palkopoulou et al. 2015 | Oymiakon (young male) | 631 M | 28 (figure S1B) | 3/12 |
| *Loxodonta africana cyclotis* | ERR2260500 | Palkopoulou et al. 2018 | Coco  (adult male) | 541 M | 41  (figure S1A) | 15/15 |
| *Mammut americanum* | ERR2260503 | Palkopoulou et al. 2018 | Unknown (male?) | 197 M | 6  (figure S1C) | 2/2 |

### Table S3: elephant specimens information and qPCR details

| Specimen | [DNA] | prior sex | Replicate | X-VIC | | Y-FAM | | Inferred |
| --- | --- | --- | --- | --- | --- | --- | --- | --- |
| ID | (ng/ul) | determination | Number | Cq | RFU | Cq | RFU | genotype |
| ELE02 | 186.7 | Male | 1 | 34.7 | 601 | 33.6 | 634 | XY |
|  |  |  | 2 | 32.5 | 575 | 31.8 | 536 | XY |
| ELE05 | na | Male | 1 | 29.7 | 589 | 29.1 | 898 | XY |
|  |  |  | 2 | 29.6 | 677 | 29.9 | 941 | XY |
| ELE06 | 55.7 | Male | 1 | 29.3 | 723 | 28.1 | 1125 | XY |
|  |  |  | 2 | 29.4 | 733 | 29.2 | 1256 | XY |
| ELE20 | 2.9 | Male | 1 | No Cq | 11.2 | No Cq | 17.1 | ? |
|  |  |  | 2 | No Cq | 34.1 | No Cq | 122 | ? |
| ELE44 | na | Male | 1 | 31.0 | 393 | 30.3 | 551 | XY |
|  |  |  | 2 | 31.4 | 504 | 34.4 | 295 | XY |
| ELE69 | 67.4 | Unknown | 1 | 37.1 | 296 | 35.4 | 630 | XY |
|  |  |  | 2 | 36.3 | 345 | 37.6 | 211 | XY |
| ELE71 | 56.5 | Male | 1 | 29.9 | 532 | 29.0 | 949 | XY |
|  |  |  | 2 | 30.3 | 593 | 30.0 | 1066 | XY |
| ELE82 | 28.7 | Female | 1 | 31.5 | 686 | No Cq | 30.1 | XX |
|  |  |  | 2 | 31.6 | 801 | No Cq | 30.2 | XX |
| ELE90 | na | Unknown | 1 | 32.5 | 749 | No Cq | 14.2 | XX |
|  |  |  | 2 | 31.9 | 834 | No Cq | 13.2 | XX |
| ELE91 | 38.3 | Female | 1 | 29.4 | 1313 | No Cq | 42.7 | XX |
|  |  |  | 2 | 29.8 | 851 | No Cq | 44.8 | XX |
| ELE92 | 35.4 | Female | 1 | 29.2 | 1325 | No Cq | 42.6 | XX |
|  |  |  | 2 | 29.6 | 953 | No Cq | 93.0 | XX |
| ELE93 | 26.9 | Female | 1 | 30.9 | 851 | No Cq | 32.3 | XX |
|  |  |  | 2 | 30.7 | 920 | No Cq | 48.3 | XX |
| ELE94 | 23.1 | Female | 1 | 31.1 | 767 | No Cq | 30.9 | XX |
|  |  |  | 2 | 31.9 | 784 | No Cq | 48.6 | XX |
| ELE95 | 34.5 | Unknown | 1 | 32.6 | 699 | No Cq | 30.6 | XX |
|  |  |  | 2 | 31.7 | 776 | No Cq | 38.8 | XX |
| ELE96 | 16.1 | Unknown | 1 | 31.4 | 808 | No Cq | 29.3 | XX |
|  |  |  | 2 | 31.1 | 799 | No Cq | 42.2 | XX |
| ELE97 | 44.6 | Unknown | 1 | 34.3 | 464 | 34.1 | 617 | XY |
|  |  |  | 2 | 34.0 | 485 | 32.8 | 908 | XY |
| ELE98 | 12.6 | Unknown | 1 | 36.3 | 147 | 35.2 | 737 | XY |
|  |  |  | 2 | 36.6 | 247 | 34.8 | 726 | XY |
| ELE99 | 56.7 | Unknown | 1 | 29.3 | 741 | 28.6 | 1041 | XY |
|  |  |  | 2 | 29.0 | 697 | 27.9 | 1169 | XY |
| ELE100 | na | Unknown | 1 | 35.3 | 441 | 35.5 | 479 | XY |
|  |  |  | 2 | 36.7 | 205 | 34.9 | 892 | XY |
| ELE101 | na | Unknown | 1 | 31.2 | 706 | 30.4 | 899 | XY |
|  |  |  | 2 | 31.3 | 681 | 30.4 | 1171 | XY |
| ELE102 | na | Unknown | 1 | 30.6 | 641 | 29.6 | 850 | XY |
|  |  |  | 2 | 30.3 | 707 | 29.4 | 1181 | XY |
| ELE103 | na | Unknown | 1 | 29.7 | 749 | 29.0 | 1023 | XY |
|  |  |  | 2 | 29.3 | 696 | 28.8 | 1311 | XY |
| ELE104 | na | Unknown | 1 | 29.9 | 727 | 29.3 | 1022 | XY |
|  |  |  | 2 | 29.0 | 711 | 28.9 | 1181 | XY |
| ELE105 | na | Unknown | 1 | 27.8 | 713 | 27.2 | 991 | XY |
|  |  |  | 2 | 28.0 | 804 | 27.7 | 1367 | XY |
| ELE106 | na | Unknown | 1 | 34.0 | 496 | 33.3 | 779 | XY |
|  |  |  | 2 | 33.7 | 433 | 33.2 | 845 | XY |
| ELE107 | na | Unknown | 1 | 36.0 | 367 | 35.5 | 606 | XY |
|  |  |  | 2 | 31.0 | 595 | 3.0 | 923 | XY |
| ELE108 | na | Unknown | 1 | 34.3 | 461 | 33.3 | 823 | XY |
|  |  |  | 2 | 32.8 | 456 | 31.9 | 1037 | XY |
| ELE109 | na | Unknown | 1 | 34.8 | 494 | 34.2 | 793 | XY |
|  |  |  | 2 | 34.6 | 458 | 34.0 | 694 | XY |
| ELE110 | na | Unknown | 1 | 34.5 | 506 | 34.4 | 669 | XY |
|  |  |  | 2 | 33.9 | 525 | 33.6 | 688 | XY |
| ELE111 | na | Unknown | 1 | 33.1 | 677 | 33.6 | 690 | XY |
|  |  |  | 2 | 33.7 | 455 | 32.5 | 888 | XY |
| ELE112 | na | Unknown | 1 | 33.7 | 575 | 34.7 | 503 | XY |
|  |  |  | 2 | 32.1 | 627 | 32.0 | 786 | XY |
| ELE113 | na | Unknown | 1 | 33.8 | 563 | 32.5 | 899 | XY |
|  |  |  | 2 | 33.8 | 485 | 32.8 | 875 | XY |
| ELE114 | na | Unknown | 1 | 34.1 | 597 | 35.1 | 449 | XY |
|  |  |  | 2 | 34.7 | 445 | 34.1 | 682 | XY |
| ELE115 | na | Female | 1 | 32.1 | 555 | No Cq | 21.6 | XX |
|  |  |  | 2 | 32.0 | 828 | No Cq | 46.2 | XX |
| ELE116 | na | Unknown | 1 | 33.1 | 669 | 32.6 | 709 | XY |
|  |  |  | 2 | 32.2 | 498 | 31.6 | 1021 | XY |
| ELE117 | na | Unknown | 1 | 33.3 | 654 | No Cq | 17.1 | XX |
|  |  |  | 2 | 33.2 | 653 | No Cq | 20.2 | XX |
| ELE118 | na | Unknown | 1 | 37.0 | 458 | 35.7 | 348 | XY |
|  |  |  | 2 | 34.8 | 388 | 35.3 | 507 | XY |
| ELE119 | na | Unknown | 1 | 29.3 | 564 | 28.7 | 753 | XY |
|  |  |  | 2 | 32.7 | 599 | 33.0 | 867 | XY |
| ELE120 | 33.6 | Unknown | 1 | 29.3 | 798 | 28.7 | 1145 | XY |
|  |  |  | 2 | 29.6 | 736 | 28.6 | 1155 | XY |
| ELE121 | 58 | Unknown | 1 | 28.9 | 778 | 28.2 | 1099 | XY |
|  |  |  | 2 | 30.1 | 724 | 28.8 | 1263 | XY |
| ELE122 | 27.1 | Unknown | 1 | 29.9 | 774 | 29.4 | 1102 | XY |
|  |  |  | 2 | 30.5 | 649 | 29.2 | 1165 | XY |
| ELE123 | na | Unknown | 1 | 31.6 | 575 | 30.6 | 938 | XY |
|  |  |  | 2 | 31.4 | 631 | 30.0 | 1182 | XY |
| ELE127 | na | Unknown | 1 | 29.8 | 672 | 28.8 | 980 | XY |
|  |  |  | 2 | 29.6 | 746 | 28.7 | 1168 | XY |
| ELE128 | na | Unknown | 1 | 32.6 | 516 | 31.2 | 841 | XY |
|  |  |  | 2 | 31.9 | 605 | 30.5 | 1130 | XY |
| ELE129 | na | Unknown | 1 | 30.5 | 643 | 29.3 | 932 | XY |
|  |  |  | 2 | 30.3 | 706 | 29.4 | 1082 | XY |
| ELE130 | na | Unknown | 1 | 32.6 | 766 | No Cq | 17.6 | XX |
|  |  |  | 2 | 32.1 | 767 | No Cq | 45.3 | XX |
| ELE131 | na | Unknown | 1 | 31.0 | 1016 | No Cq | 39.5 | XX |
|  |  |  | 2 | 30.6 | 803 | No Cq | 50.3 | XX |
| ELE132 | na | Unknown | 1 | 28.5 | 804 | 27.8 | 1179 | XY |
|  |  |  | 2 | 27.9 | 750 | 27.3 | 1368 | XY |
| ELE133 | 8.3 | Unknown | 1 | 36.9 | 438 | 36.1 | 374 | XY |
|  |  |  | 2 | 36.2 | 499 | 35.5 | 569 | XY |
| ELE135 | 15.1 | Unknown | 1 | 34.6 | 465 | 33.4 | 950 | XY |
|  |  |  | 2 | 34.5 | 454 | 33.2 | 901 | XY |
| ELE136 | 13.7 | Unknown | 1 | 35.8 | 369 | 34.5 | 826 | XY |
|  |  |  | 2 | 35.9 | 362 | 35.5 | 513 | XY |
| ELE136B | na | Unknown | 1 | 33.5 | 542 | 33.4 | 686 | XY |
|  |  |  | 2 | 33.8 | 493 | 34.1 | 719 | XY |
| ELE137 | 23.0 | Unknown | 1 | 32.7 | 696 | 32.4 | 927 | XY |
|  |  |  | 2 | 33.1 | 545 | 32.1 | 1008 | XY |
| ELE138 | na | Unknown | 1 | 34.5 | 581 | 35.0 | 596 | XY |
|  |  |  | 2 | 35.7 | 359 | 34.9 | 644 | XY |
| ELE139 | 8.5 | Unknown | 1 | 31.4 | 723 | 30.7 | 1051 | XY |
|  |  |  | 2 | 32.1 | 577 | 30.6 | 1132 | XY |
| ELE140 | 5.7 | Unknown | 1 | 35.6 | 472 | 34.6 | 768 | XY |
|  |  |  | 2 | 32.8 | 536 | 33.5 | 575 | XY |
| ELE141 | 49.3 | Unknown | 1 | 34.8 | 553 | 34.5 | 643 | XY |
|  |  |  | 2 | 35.3 | 389 | 34.3 | 734 | XY |
| ELE142 | 27.7 | Unknown | 1 | 35.3 | 376 | 34.2 | 657 | XY |
|  |  |  | 2 | 33.3 | 530 | 34.8 | 444 | XY |
| ELE143 | 43.5 | Unknown | 1 | 30.1 | 678 | 29.3 | 1006 | XY |
|  |  |  | 2 | 29.1 | 681 | 28.6 | 1241 | XY |
| ELE144 | 16.4 | Unknown | 1 | 34.5 | 468 | 33.4 | 886 | XY |
|  |  |  | 2 | 34.0 | 345 | 33.6 | 714 | XY |
| ELE145 | 38.2 | Unknown | 1 | 34.1 | 572 | 33.6 | 853 | XY |
|  |  |  | 2 | 33.1 | 534 | 33.4 | 805 | XY |
| ELE146 | 26.7 | Unknown | 1 | 33.1 | 635 | 32.5 | 949 | XY |
|  |  |  | 2 | 32.0 | 562 | 31.5 | 1061 | XY |
| ELE147 | 14.7 | Unknown | 1 | 33.1 | 683 | 32.7 | 938.5 | XY |
|  |  |  | 2 | 32.7 | 598 | 31.5 | 1103 | XY |
| ELE148 | 18.5 | Unknown | 1 | 36.4 | 439 | No Cq | 16.4 | XX |
|  |  |  | 2 | 38.1 | 182 | No Cq | 5.1 | XX |
| ELE149 | 8.6 | Unknown | 1 | 36.2 | 387 | 36.1 | 526 | XY |
|  |  |  | 2 | 30.7 | 697 | 30.2 | 963 | XY |
| ELE150 | 18.3 | Unknown | 1 | 32.7 | 738 | 33.2 | 796 | XY |
|  |  |  | 2 | 33.1 | 576 | 32.0 | 1063 | XY |
| ELE151 | 19.0 | Unknown | 1 | 34.6 | 656 | No Cq | 18.5 | XX |
|  |  |  | 2 | 34.2 | 675 | No Cq | 30.7 | XX |
| ELE152 | 59.1 | Unknown | 1 | 27.6 | 1140 | No Cq | 9.5 | XX |
|  |  |  | 2 | 27.4 | 1165 | No Cq | 25.6 | XX |
| ELE153 | 59.9 | Unknown | 1 | 29.9 | 959 | No Cq | 40.2 | XX |
|  |  |  | 2 | 29.6 | 1055 | No Cq | 103 | XX |
| ELE154 | 11.1 | Unknown | 1 | 35.7 | 388 | 35.3 | 525 | XY |
|  |  |  | 2 | 36.2 | 205 | 34.9 | 620 | XY |
| ELE155 | 27.5 | Unknown | 1 | 35.1 | 426 | 34.3 | 737 | XY |
|  |  |  | 2 | 33.7 | 417 | 33.8 | 646 | XY |
| ELE156 | 20.9 | Unknown | 1 | 33.4 | 491 | 32.2 | 929 | XY |
|  |  |  | 2 | 33.3 | 381 | 31.9 | 1098 | XY |
| ELE157 | 15.4 | Unknown | 1 | 33.6 | 503 | 32.7 | 855 | XY |
|  |  |  | 2 | 33.3 | 371 | 32.7 | 815 | XY |
| ELE158 | 29.0 | Unknown | 1 | 35.4 | 548 | 34.1 | 682 | XY |
|  |  |  | 2 | 30.1 | 687 | 29.6 | 967 | XY |
| ELE159 | 27.1 | Unknown | 1 | 35.0 | 582 | No Cq | 17.0 | XX |
|  |  |  | 2 | 35.6 | 474 | No Cq | 26.7 | XX |
| ELE160 | 23.6 | Unknown | 1 | 34.8 | 470 | 34.0 | 801 | XY |
|  |  |  | 2 | 34.4 | 517 | 33.6 | 837 | XY |
| ELE161 | 37.4 | Unknown | 1 | 32.5 | 834 | No Cq | 35.2 | XX |
|  |  |  | 2 | 32.7 | 860 | No Cq | 49.1 | XX |
| ELE162 | 45.7 | Unknown | 1 | 35.1 | 434 | 35.2 | 497 | XY |
|  |  |  | 2 | 34.3 | 523 | 34.3 | 628 | XY |
| ELE163 | 29.8 | Unknown | 1 | 32.9 | 612 | 32.5 | 811 | XY |
|  |  |  | 2 | 32.9 | 656 | 32.2 | 990 | XY |
| ELE164 | 51.2 | Unknown | 1 | 34.0 | 539 | 33.6 | 641 | XY |
|  |  |  | 2 | 32.1 | 697 | 32.1 | 874 | XY |
| ELE165 | 29.1 | Unknown | 1 | 34.2 | 480 | 33.3 | 710 | XY |
|  |  |  | 2 | 33.0 | 584 | 32.2 | 969 | XY |
| ELE166 | 65.9 | Unknown | 1 | 32.0 | 789 | No Cq | 14.8 | XX |
|  |  |  | 2 | 31.3 | 757 | No Cq | 39.5 | XX |
| ELE167 | 29.6 | Unknown | 1 | 31.3 | 794 | No Cq | 15.8 | XX |
|  |  |  | 2 | 30.5 | 860 | No Cq | 47.3 | XX |
| ELE168 | 14.1 | Unknown | 1 | 35.6 | 440 | No Cq | 14.3 | XX |
|  |  |  | 2 | 35.0 | 781 | No Cq | 14.1 | XX |
| ELE169 | 12.6 | Unknown | 1 | 35.4 | 373 | 34.1 | 829 | XY |
|  |  |  | 2 | 33.8 | 760 | 34.1 | 1096 | XY |
| ELE170 | 19.7 | Unknown | 1 | 33.4 | 585 | 33.5 | 718 | XY |
|  |  |  | 2 | 32.2 | 530 | 31.7 | 1043 | XY |
| ELE171 | 50.1 | Unknown | 1 | 28.4 | 1120 | No Cq | 40.7 | XX |
|  |  |  | 2 | 28.4 | 1044 | No Cq | 58.5 | XX |
| ELE172 | 59.1 | Unknown | 1 | 32.5 | 845 | No Cq | 20.5 | XX |
|  |  |  | 2 | 32.3 | 864 | No Cq | 93 | XX |
| ELE173 | 63.3 | Unknown | 1 | 30.2 | 966 | No Cq | 13.2 | XX |
|  |  |  | 2 | 30.1 | 1042 | No Cq | 58.3 | XX |
| ELE174 | 26.4 | Unknown | 1 | 32.7 | 809 | No Cq | 23.3 | XX |
|  |  |  | 2 | 33.0 | 844 | No Cq | 46.8 | XX |
| ELE175 | 34.3 | Unknown | 1 | 29.2 | 992 | No Cq | 11.6 | XX |
|  |  |  | 2 | 28.7 | 1138 | No Cq | 67.4 | XX |
| ELE176 | 20.0 | Unknown | 1 | 32.1 | 949 | No Cq | 20.7 | XX |
|  |  |  | 2 | 32.1 | 922 | No Cq | 55.5 | XX |
| ELE177 | 36.9 | Unknown | 1 | 32.5 | 877 | No Cq | 35.7 | XX |
|  |  |  | 2 | 32.9 | 838 | No Cq | 58.6 | XX |
| ELE178 | 34.0 | Unknown | 1 | 32.1 | 530 | 30.9 | 917 | XY |
|  |  |  | 2 | 30.7 | 595 | 30.3 | 1136 | XY |
| ELE179 | 39.8 | Unknown | 1 | 29.6 | 596 | 29.0 | 890 | XY |
|  |  |  | 2 | 29.0 | 700 | 29.9 | 1155 | XY |
| ELE180 | 15.4 | Unknown | 1 | 31.6 | 809 | No Cq | 18.5 | XX |
|  |  |  | 2 | 31.9 | 786 | No Cq | 94.0 | XX |
| ELE181 | 100.6 | Unknown | 1 | 29.0 | 661 | 28.2 | 951 | XY |
|  |  |  | 2 | 28.6 | 668 | 28.3 | 1171 | XY |
| ELE182 | 27.6 | Unknown | 1 | 33.7 | 613 | No Cq | 15.6 | XX |
|  |  |  | 2 | 33.9 | 549 | No Cq | 9.9 | XX |
| ELE183 | 24.3 | Unknown | 1 | 32.0 | 807 | No Cq | 20.9 | XX |
|  |  |  | 2 | 32.0 | 845 | No Cq | 31.5 | XX |
| ELE184 | 17.6 | Unknown | 1 | 30.4 | 847 | No Cq | 12.2 | XX |
|  |  |  | 2 | 30.1 | 996 | No Cq | 61.9 | XX |
| ELE185 | 28.7 | Unknown | 1 | 34.0 | 551 | 34.9 | 483 | XY |
|  |  |  | 2 | 34.2 | 497 | 33.7 | 755 | XY |
| ELE186 | 24.5 | Unknown | 1 | 31.3 | 538 | 30.2 | 836 | XY |
|  |  |  | 2 | 31.0 | 700 | 30.0 | 1170 | XY |
| ELE187 | 22.1 | Unknown | 1 | 33.7 | 594 | 33.5 | 685 | XY |
|  |  |  | 2 | 33.0 | 545 | 33.0 | 879 | XY |
| NTC | - | - | 1 | No Cq | -0.3 | No Cq | -0.4 | PCR- |
|  |  |  | 2 | No Cq | -1.7 | No Cq | -0.6 | PCR- |
|  |  |  | 3 | No Cq | -0.5 | No Cq | -1.1 | PCR- |
|  |  |  | 4 | No Cq | -1.1 | No Cq | 0.2 | PCR- |
|  |  |  | 5 | No Cq | 0.9 | No Cq | -2.1 | PCR- |
|  |  |  | 6 | No Cq | -1.3 | No Cq | -1.8 | PCR- |
|  |  |  | 7 | No Cq | -1.3 | No Cq | -2 | PCR- |
|  |  |  | 8 | No Cq | -1 | No Cq | -0.9 | PCR- |
|  |  |  | 9 | No Cq | 0.2 | No Cq | -1.9 | PCR- |

### Table S4 : mammoth specimens information and qPCR details

| Specimen | 14C | Reference | [DNA] | anatomical | Replicate | X-VIC | | Y-FAM | | Inferred |
| --- | --- | --- | --- | --- | --- | --- | --- | --- | --- | --- |
| ID | date |  | (ng/ul) | sex | Number | CN | RFU | CN | RFU | genotype |
| Khroma | > 50,000 | this study | 8.9 | Female | 1 | 1035.7 | 1124 | 0 | 14 | XX |
|  |  |  |  |  | 2 | 827.1 | 899 | 0 | 24 | XX |
|  |  |  |  |  | 3 | 799.8 | 875 | 0 | 31 | XX |
| 2005/931 | - | this study | 6.3 | unknown | 1 | 967 | 1120 | 0 | 28 | XX |
|  |  |  |  |  | 2 | 458.6 | 544 | 0 | 14 | XX |
|  |  |  |  |  | 3 | 609.4 | 616 | 0 | 26 | XX |
| 2005/918 | - | this study | 7.7 | unknown | 1 | 876 | 1146 | 0 | 25 | XX |
|  |  |  |  |  | 2 | 291.5 | 546 | 0 | 20 | XX |
|  |  |  |  |  | 3 | 333.5 | 516 | 0 | 34 | XX |
| 2005/898 | - | 2008* | 6.5 | unknown | 1 | 334.6 | 860 | 299.9 | 1093 | XY |
|  |  |  |  |  | 2 | 221.2 | 467 | 308.3 | 793 | XY |
|  |  |  |  |  | 3 | 88.5 | 367 | 103.4 | 591 | XY |
| Oymiakon | 44,800 | 2008 | 7.2 | Male | 1 | 370.7 | 780 | 315 | 1000 | XY |
|  | ± 1,010 |  |  |  | 2 | 168.6 | 367 | 107 | 689 | XY |
|  |  |  |  |  | 3 | 150.4 | 348 | 101.6 | 626 | XY |
| 2005/897 | 40,150 | 2008 | 7.4 | unknown | 1 | 199 | 844 | 152 | 1029 | XY |
|  | ± 990 |  |  |  | 2 | 146.2 | 467 | 184.6 | 823 | XY |
|  |  |  |  |  | 3 | 60.9 | 415 | 94.5 | 770 | XY |
| Lyakhov | > 50,000 | 2003** | 6.5 | Male | 1 | 156.7 | 884 | 119.1 | 1034 | XY |
|  |  |  |  |  | 2 | 166.5 | 642 | 131.3 | 882 | XY |
|  |  |  |  |  | 3 | 132.3 | 515 | 112.7 | 822 | XY |
| 2005/915 | 27,740 | 2008 | 5.4 | unknown | 1 | 161.1 | 806 | 121.9 | 931 | XY |
|  | ± 220 |  |  |  | 2 | 57.6 | 368 | 77.9 | 613 | XY |
|  |  |  |  |  | 3 | 40.2 | 355 | 43.2 | 557 | XY |
| 2005/924 | - | 2008 | 5.0 | unknown | 1 | 29.7 | 681 | 34.9 | 1020 | XY |
|  |  |  |  |  | 2 | 41.3 | 598 | 44.2 | 797 | XY |
|  |  |  |  |  | 3 | 33.6 | 662 | 73.4 | 813 | XY |
| WR2 | 4,420 | 2008 | 3.2 | unknown | 1 | 61.4 | 907 | 0 | 27 | XX |
|  | ± 15 |  |  |  | 2 | 52.6 | 496 | 0 | 19 | XX |
|  |  |  |  |  | 3 | 46 | 429 | 0 | 30 | XX |
| 2000/174 | 28,210 | 2008 | 2.8 | Female | 1 | 26.7 | 615 | 0 | 45 | XX |
|  | ± 210 |  |  |  | 2 | 29.6 | 344 | 0 | 24 | XX |
|  |  |  |  |  | 3 | 18.1 | 201 | 0 | 11 | XX |
| 2005/999 | > 49,900 | 2008 | 4.5 | unknown | 1 | 42.1 | 760 | 0 | 17 | XX |
|  |  |  |  |  | 2 | 10.2 | 360 | 0 | 14 | XX |
|  |  |  |  |  | 3 | 14.1 | 389 | 0 | 15 | XX |
| 2005/913 | - | this study | 2.3 | unknown | 1 | 3.6 | 274 | 3.2 | 442 | XY |
|  |  |  |  |  | 2 | 10 | 300 | 18 | 616 | XY |
|  |  |  |  |  | 3 | 7.8 | 272 | 19.5 | 659 | XY |
| 2005/900 | 28,700 | 2008 | 4.4 | unknown | 1 | 20.1 | 671 | 0 | 20 | XX |
|  | ± 310 |  |  |  | 2 | 3.7 | 271 | 0 | 16 | XX |
|  |  |  |  |  | 3 | 7.2 | 304 | 0 | 13 | XX |
| Jarkov | 20,390 | 2008 | 3.7 | Male | 1 | 5.6 | 314 | 2.8 | 365 | XY |
|  | ± 160 |  |  |  | 2 | 2.7 | 245 | 3.1 | 323 | XY |
|  |  |  |  |  | 3 | 2.9 | 200 | 3 | 312 | XY |
| Lyuba | 41,700 | this study | 4.1 | Female | 1 | 1.7 | 206 | 0 | 78 | XX |
|  | ± 700 |  |  |  | 2 | 2.1 | 330 | 0 | 13 | XX |
|  |  |  |  |  | 3 | 6 | 415 | 0 | 25 | XX |
| 2005/945 | 20,080 | 2008 | 3.7 | unknown | 1 | 1.3 | 153 | 3.4 | 560 | XY |
|  | ± 110 |  |  |  | 2 | 0.7 | 126 | 0.5 | 109 | XY |
|  |  |  |  |  | 3 | 0 | 7.3 | 2.3 | 370 | YY |
| 2001/451 | - | 2008 | 3.5 | unknown | 1 | 3.3 | 362 | 0 | 13 | XX |
|  |  |  |  |  | 2 | 1.3 | 211 | 0 | 12 | XX |
|  |  |  |  |  | 3 | 0 | -0.4 | 0 | -1.8 | PCR- |
| 2002/489 | - | this study | 3.3 | unknown | 1 | 1.4 | 163 | 1.9 | 389 | XY |
|  |  |  |  |  | 2 | 1.1 | 152 | 0.3 | 83 | XY |
|  |  |  |  |  | 3 | 0.3 | 64 | 0.4 | 96 | XY |
| 2003/838 | - | this study | 1.2 | unknown | 1 | 1.9 | 202 | 0.3 | 107 | ? |
|  |  |  |  |  | 2 | 0 | 0.0 | 0.9 | 227 | YY |
|  |  |  |  |  | 3 | 0 | -1.2 | 0 | 3.6 | PCR- |
| 2005/927 | - | this study | 1.4 | unknown | 1 | 1.9 | 254 | 0 | 7.2 | XX |
|  |  |  |  |  | 2 | 1.1 | 149 | 0.4 | 103 | XY |
|  |  |  |  |  | 3 | 1.2 | 183 | 0.3 | 84 | XY |
| 2000/165 | - | this study | 0.8 | unknown | 1 | 1.5 | 203 | 0 | 9.0 | XX |
|  |  |  |  |  | 2 | 0.7 | 125 | 0 | 5.4 | XX |
|  |  |  |  |  | 3 | 1.2 | 216 | 1 | 136 | XY |
| 2000/187 | - | this study | 1.3 | unknown | 1 | 1.4 | 183 | 0 | 5.0 | XX |
|  |  |  |  |  | 2 | 0 | 0.7 | 1 | 251 | YY |
|  |  |  |  |  | 3 | 0.7 | 73 | 1.4 | 370 | XY |
| 2005/904 | - | this study | 0.5 | unknown | 1 | 0.6 | 46 | 0.9 | 200 | XY |
|  |  |  |  |  | 2 | 0.6 | 106 | 0.8 | 80 | XY |
|  |  |  |  |  | 3 | 0.9 | 122 | 0.3 | 73 | XY |
| 2000/175 | - | this study | 0.6 | unknown | 1 | 1.1 | 339 | 0 | 13 | XX |
|  |  |  |  |  | 2 | 1.2 | 223 | 0 | 7.1 | XX |
|  |  |  |  |  | 3 | 0 | 3.9 | 0 | 1.4 | PCR- |
| 2000/176 | - | this study | 0.9 | unknown | 1 | 0.9 | 114 | 0 | 2.0 | ? |
|  |  |  |  |  | 2 | 0.2 | 48 | 0 | 1.3 | ? |
|  |  |  |  |  | 3 | 0 | 0.4 | 0 | -1.1 | PCR- |
| 2002/472 | > 48,800 | 2008 | 2.7 | unknown | 1 | 0 | -0.9 | 0 | -0.3 | PCR- |
|  |  |  |  |  | 2 | 0 | 16.2 | 0 | 5.2 | PCR- |
|  |  |  |  |  | 3 | 0 | -0.5 | 0 | -2.5 | PCR- |
| 2002/473 | 46,700 | 2008 | 1.2 | unknown | 1 | 0 | 14.2 | 0 | 4.1 | PCR- |
|  | ± 2800 |  |  |  | 2 | 0 | 12.0 | 0 | 3.3 | PCR- |
|  |  |  |  |  | 3 | 0 | 1.3 | 0 | -0.1 | PCR- |
| 2005/1000 | - | 2008 | 0.9 | unknown | 1 | 0 | -0.6 | 0 | -0.2 | PCR- |
|  |  |  |  |  | 2 | 0 | 30.7 | 0 | 6.1 | PCR- |
|  |  |  |  |  | 3 | 0 | -1.2 | 0 | -1.8 | PCR- |
| E. blank 1 | - | - | < 0.05 | - | 1 | 0 | 4.1 | 0 | 2.0 | PCR- |
|  |  |  |  |  | 2 | 0 | 3.9 | 0 | 1.1 | PCR- |
|  |  |  |  |  | 3 | 0 | 0.8 | 0 | -0.9 | PCR- |
| E. blank 2 | - | - | < 0.05 | - | 1 | 0 | 0.6 | 0 | 0.5 | PCR- |
|  |  |  |  |  | 2 | 0 | -0.1 | 0 | -4.1 | PCR- |
|  |  |  |  |  | 3 | 0 | 1.5 | 0 | 1.3 | PCR- |
| E. blank 3 | - | - | < 0.05 | - | 1 | 0 | 1.1 | 0 | 0.9 | PCR- |
|  |  |  |  |  | 2 | 0 | 0.3 | 0 | 1.7 | PCR- |
|  |  |  |  |  | 3 | 0 | -0.5 | 0 | 0.0 | PCR- |
| E. blank 4 | - | - | < 0.05 | - | 1 | 0 | 1.7 | 0 | 2.8 | PCR- |
|  |  |  |  |  | 2 | 0 | -0.9 | 0 | -0.7 | PCR- |
|  |  |  |  |  | 3 | 0 | 1.2 | 0 | 1.1 | PCR- |
| E. blank 5 | - | - | < 0.05 | - | 1 | 0 | 0.8 | 0 | 0.2 | PCR- |
|  |  |  |  |  | 2 | 0 | -0.5 | 0 | -0.3 | PCR- |
|  |  |  |  |  | 3 | 0 | 1.5 | 0 | 3.4 | PCR- |
| NTC | - | - | - | - | 1 | 0 | -0.3 | 0 | -3.0 | PCR- |
|  |  |  |  |  | 2 | 0 | -0.6 | 0 | -2.8 | PCR- |
|  |  |  |  |  | 3 | 0 | -0.8 | 0 | -1.3 | PCR- |
|  |  |  |  |  | 4 | 0 | 1.0 | 0 | -2.7 | PCR- |
|  |  |  |  |  | 5 | 0 | -1.3 | 0 | 0.9 | PCR- |
|  |  |  |  |  | 6 | 0 | -1.1 | 0 | -3.4 | PCR- |
|  |  |  |  |  | 7 | 0 | -0.6 | 0 | 3.6 | PCR- |

* Debruyne et al. 2008 (see main text)
** Debruyne et al. 2003 (see main text)

### Table S5: Quantification standard results (quantification cycle Cq) via total SYBR green quantification of the standard series

| Copy number | Replicate number/total | Cq |
| --- | --- | --- |
| 2x10^5 | 1/1 | 20.2 |
| 2x10^4 | 1/1 | 23.5 |
| 2x10^3 | 1/1 | 26.5 |
| 2x10^2 | 1/1 | 30.0 |
| 2x10^1 | 1/1 | 33.2 |
| 2x10^0 | 1/6 | 35.8 |
| 2x10^0 | 2/6 | 36.9 |
| 2x10^0 | 3/6 | 36.0 |
| 2x10^0 | 4/6 | 37.0 |
| 2x10^0 | 5/6 | 36.1 |
| 2x10^0 | 6/6 | 36.8 |
| 2x10^-1 | 1/6 | No Cq |
| 2x10^-1 | 2/6 | 37.0 |
| 2x10^-1 | 3/6 | No Cq |
| 2x10^-1 | 4/6 | No Cq |
| 2x10^-1 | 5/6 | No Cq |
| 2x10^-1 | 6/6 | No Cq |
| NTC | 1/1 | No Cq |

### Table S6: Details of calculation for the probability (P_XX_)^n^ of Y allele dropout for mammoths specimens, based on the model by Taberlet et al. 1996 (see main text and Supplemental Fig. S4). Only specimens for which (P_XX_)^n^ > 0.1% are shown.

| Specimen | Number n  of PCR+ | Total CN per reaction | U estimate (CN/2) | P_XY_ | P_XX_ | (P_XX_)^n^ |
| --- | --- | --- | --- | --- | --- | --- |
| Lyuba | 3 | 3.3 | 1.65 | 0.681 | 0.159 | 0.004 |
| 2005/945 | 3 | 2.8 | 1.4 | 0.621 | 0.189 | 0.007 |
| 2001/451 | 2 | 2.3 | 1.15 | 0.549 | 0.225 | 0.051 |
| 2002/489 | 3 | 1.8 | 0.9 | 0.464 | 0.268 | 0.019 |
| 2003/838 | 1 | 1.6 | 0.8 | 0.426 | 0.287 | 0.287 |
| 2005/927 | 3 | 1.6 | 0.8 | 0.426 | 0.287 | 0.024 |
| 2000/165 | 2 | 1.5 | 0.75 | 0.405 | 0.297 | 0.088 |
| 2000/187 | 3 | 1.5 | 0.75 | 0.405 | 0.297 | 0.026 |
| 2005/904 | 3 | 1.4 | 0.7 | 0.384 | 0.308 | 0.029 |
| 2000/175 | 2 | 1.2 | 0.6 | 0.340 | 0.330 | 0.109 |

# Supplementary Figures

### Figure S1: alignment of ZFX/Y reads. A: African elephant. B: woolly mammoth. C: American mastodon

**A**


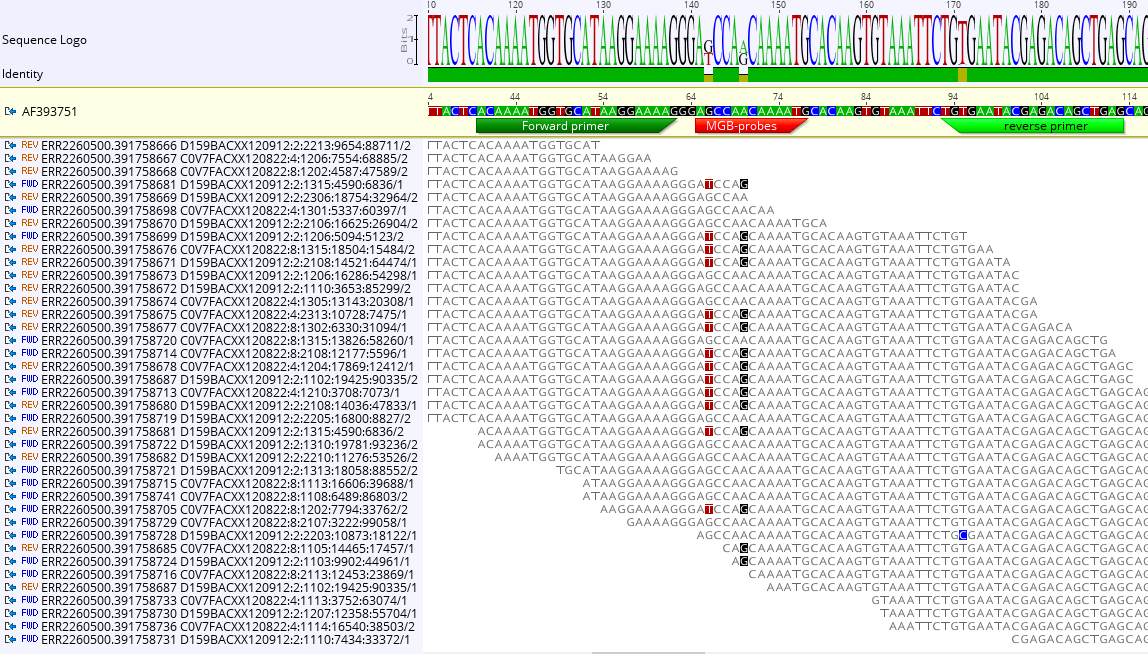


**B**


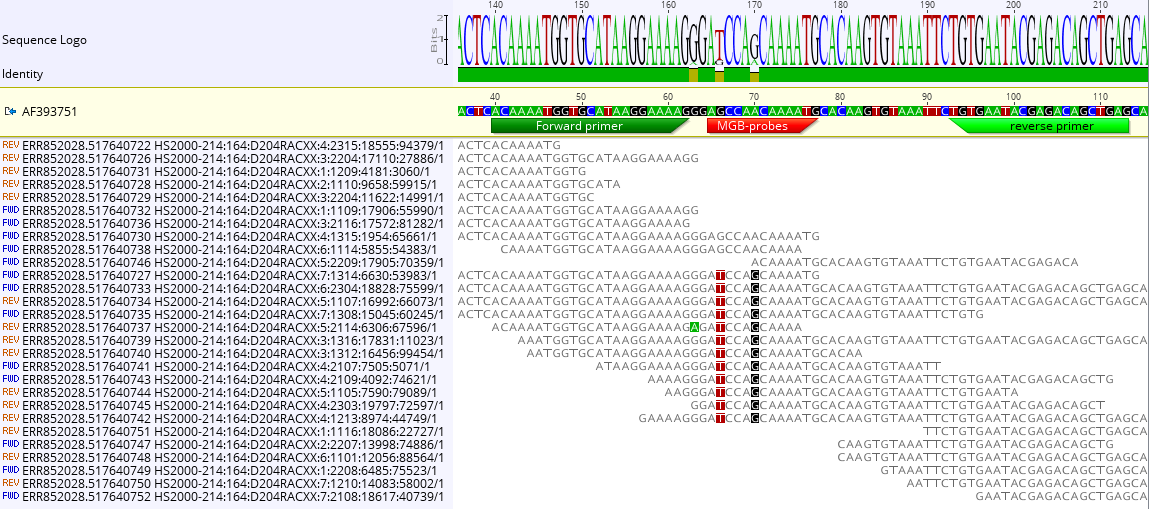


**C**


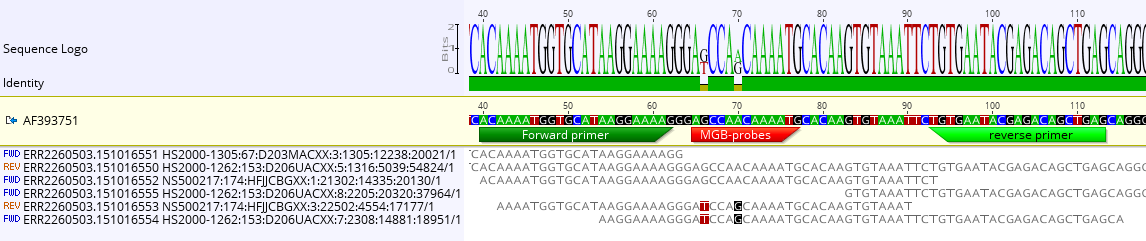


### Figure S2: closest primer and probe matches detected with potential sympatric species of elephantine taxa.


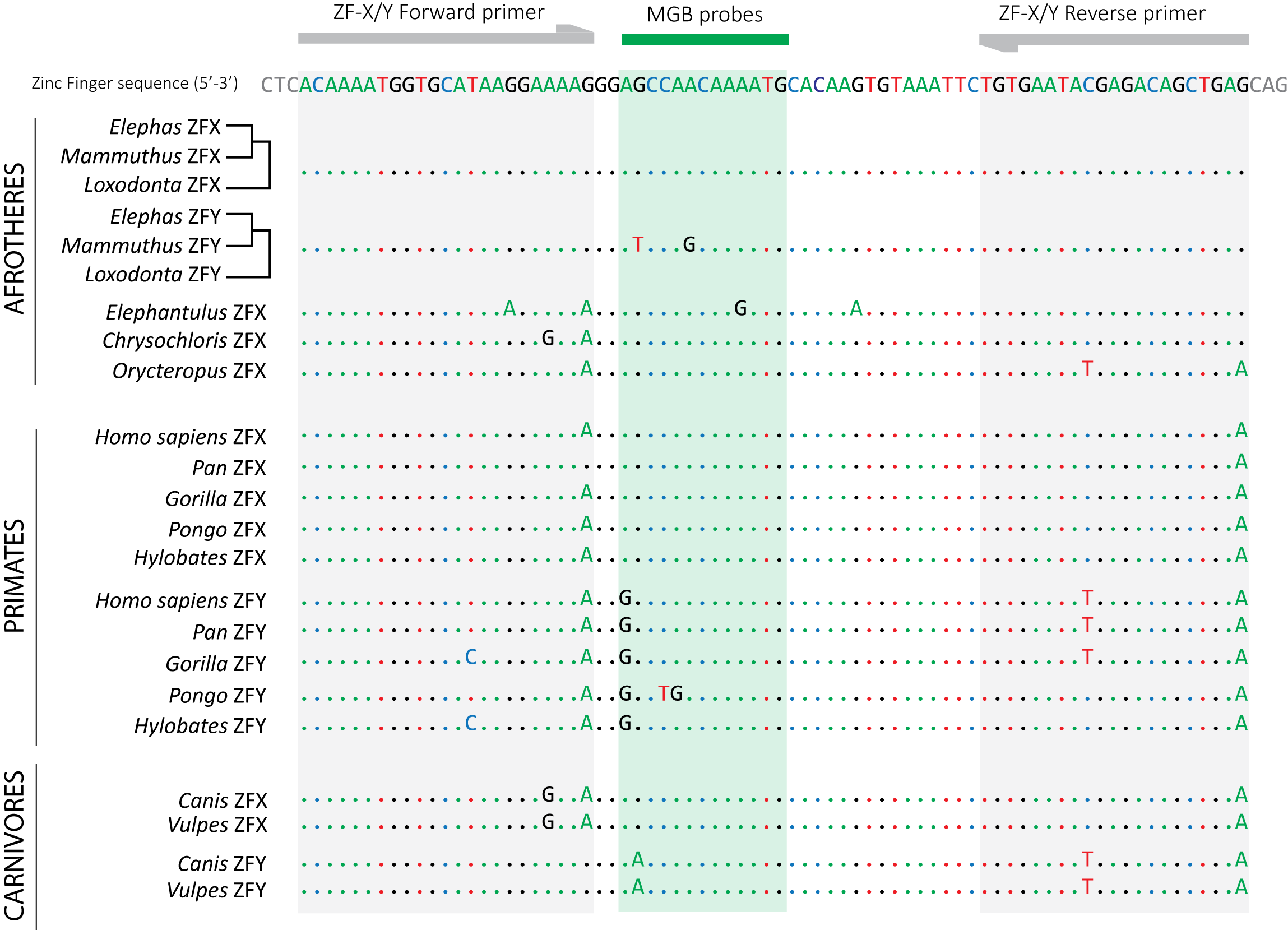


### Figure S3: Amplification plots of the sensitivity test standard series implemented in the MGB-probe assay.

###
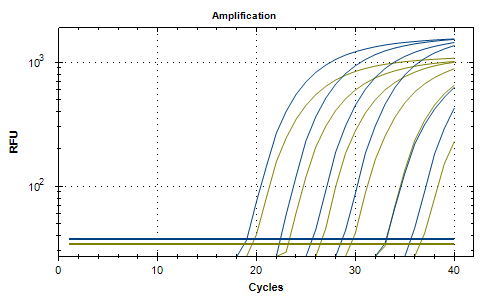


### Figure S4: Simulation results of the probability to detect the (true) heterozygous genotype (in blue) or any (false) homozygous genotype (in red) from a single PCR reaction for a bi-allelic marker; after the Fig.2 in Taberlet et al. 1996 (see main text). U: number of copies of diploid genomes per reaction. In the present case, the red distribution describes to the probability P_XX_ of erroneously genotyping a male (XY) as female (XX) in a single PCR reaction due to allelic dropout.

###
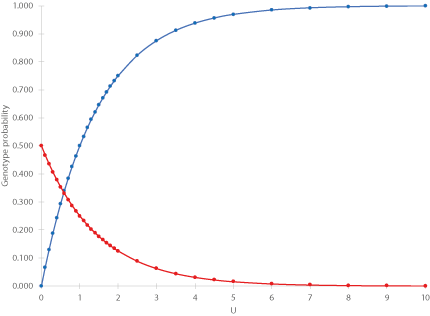

Supplement: Supplementary file 1 — Supplementary Informations. [file 41598_2021_86010_MOESM1_ESM.docx]
